# Preliminary biomechanical cadaver study investigating a new load-sharing knee implant

**DOI:** 10.1186/s40634-021-00379-2

**Published:** 2021-08-14

**Authors:** Mehdi Saeidi, Piaras A. Kelly, Christian Netzel, Miriam Scadeng, Pranesh Kumar, Deborah Prendergast, Thomas Neitzert, Maziar Ramezani

**Affiliations:** 1 Department of Mechanical Engineering, Auckland University of Technology, Auckland, New Zealand; 2 Department of Engineering Science, University of Auckland, Auckland, New Zealand; 3 Centre for Advanced Composite Materials, Department of Mechanical Engineering, University of Auckland, Auckland, New Zealand; 4 Department of Anatomy and Medical Imaging, Faculty of Health and Medical Sciences, University of Auckland, Auckland, New Zealand; 5 Centre for Functional MRI, Department of Radiology, University of California, San Diego, USA; 6 Department of Orthopaedic Surgery, Whanganui Hospital, Whanganui, New Zealand

**Keywords:** Cadaver, Extra-articular, Implant, Knee, Osteoarthritis, Tibiofemoral

## Abstract

**Purpose:**

One of the major contributors to the progression of knee osteoarthritis (OA) is the condition of loading in the knee joint. Innovatively designed load-sharing implants may be effective in terms of reducing joint load. The effects of these implants on contact joint mechanics can be evaluated through cadaver experiments. In this work, a case study is carried out with cadaver knee specimens to carry out a preliminary investigation into a novel load-sharing knee implant, in particular to study the surgical procedures required for attachment, and to determine the contact pressures in the joint with and without the implant.

**Methods:**

Contact pressure in the tibiofemoral joint was measured using pressure mapping sensors, with and without the implant, and radiographs were conducted to investigate the influence of the implant on joint space. The implant was designed from a 3D model of the specimen reconstructed by segmenting MR images of the knee, and it was manufactured by CNC machining.

**Results:**

It was observed that attachment of the implant does not affect the geometry of the hard/soft tissues. Radiographs showed that the implant led to an increase in the joint space on the medial side. Contact pressure measurements showed that the implant reduced the load on the medial side by approximately 18% under all tested loading conditions. By increasing the load from 800 to 1600 N, the percentage of load reduction in the lateral side was decreased by 8%. After applying 800, 1200, and 1600 N load it was observed that the peak contact pressures were 3.7, 4.6, and 5.5 MPa, respectively.

**Conclusions:**

This new knee implant shows some promise as a treatment for OA, through its creation of a conducive loading environment in the knee joint, without sacrificing or damaging any of the hard or soft tissues. This device could be as effective as, for example, the Atlas® system, but without some complications seen with other devices; this would need to be validated through similar results being observed in an appropriate in vivo study.

## Introduction

Knee osteoarthritis (OA) is one of the major causes of musculoskeletal impairment in adults. Initiation and progression of the OA pathology are largely associated with excessive and aberrant knee loading; however, other factors, such as trauma and genetics would be influential as well. This disease is mainly characterised by progressive degeneration of the articular cartilage and, to date, there is no known cure for knee osteoarthritis [10, 11, 15, 18]. It is believed that damage to the cartilage induced by repetitive loading, as well as an altered joint condition due to injuries, contribute to knee OA, particularly in younger individuals. For instance, one out of two individuals with a previous knee injury, such as meniscal damage or an Anterior Cruciate Ligament (ACL) rupture, develops knee osteoarthritis 10 to 20 years following the injury. Accordingly, the occurrence of those injuries in younger adults may lead to osteoarthritis when they are in their 30s or 40s [19].

For younger active patients with knee OA, common treatments include non-invasive options, i.e., weight loss, exercise, braces, physical therapy as well as pharmacologic recourses such as analgesics and anti-inflammatories [1]. These can be used to manage symptoms before considering, as a last resort, the surgical options, which are mainly recommended for patients over the age of 65 [1, 21]. General surgical options to treat knee osteoarthritis include Unicompartmental Knee Arthroplasty (UKA), High Tibial Osteotomy (HTO), and Total Knee Arthroplasty (TKA) as well as recently developed surgical interventions, such as the Atlas® system (Moximed, Inc.), which is a unicompartmental load-sharing implant [8, 9]. Among all patients who undergo knee replacement, the risk of implant failure is much higher in younger patients. For instance, a study showed that when patients under 50 years underwent knee arthroplasty, risk of two-year revision due to aseptic loosening or infection was almost four times more than for those who were over 65 at the time of the surgery [14, 18]. Also, according to some research, dissatisfaction rates after arthroplasty is higher in younger patients [6].

Although there are various options for knee OA, some physicians and patients have perceived the lack of a suitable option in between symptom management and invasive treatments [5]. According to some surveys, not only do many health care professionals recognize a treatment gap for early-onset knee osteoarthritis, but also patients would prefer to postpone the surgery until their symptoms worsen [5]. Even for patients, and particularly younger ones who are willing to undergo surgery, preference is for a method in which no bone resection is required [12, 18]. Apart from the Atlas® system, there is another innovative load-sharing implant, which has been developed recently and may fill the aforementioned gap between the conservative and invasive treatments [7, 16]. The concept of this implant is that it is extra-articular implant, comprised of femoral and tibial parts, which removes excessive load through the knee joint by attachment to the medial side. This implant would be suitable for early-onset knee osteoarthritis and can be used for younger active patients, as no major modification in the knee joint is required to attach this implant [3]. According to a few preliminary experimental and numerical studies conducted recently, this implant could be a promising option for patients with early to moderate grades of knee osteoarthritis [2, 16, 17]. The main differences between a number of available solutions, such as the Atlas® system, and the device studied here, as well as some further information on this implant concept is given in the previous numerical studies conducted by Saeidi et al. [16, 17]. Many of the complications and adverse events seen with such devices, for example clicking and squeaking noises, cracking in the knee and fracture, are likely due to the mechanical structure of the devices, skin atrophy and tissue irritation, as a result of extra-capsular installation and adjacency to soft tissue medially and laterally [13]. Given the differences in mechanical structure of the device proposed here, its intra-capsular nature, and medial attachment to the bone with the possibility of osseointegration, the authors believe that the aforementioned adverse events are less likely, or not likely, to occur.

Although computer simulations can considerably reduce cost of the development and evaluation of orthopaedic devices, experimental studies are very helpful in evaluating surgical procedures of a new prosthesis before carrying out in vivo investigations. To the best of our knowledge, no accurate experimental research has been performed to study the effect of the studied implant on the contact pressure of the tibiofemoral joint. The research reported here aims to study the surgical procedure of the implant attachment and investigate the influence of this implant on the contact pressure of a cadaver knee joint under different loading scenarios.

## Materials and methods

### Implant design and manufacturing

Knee joint specimens for this study were dissected from the left legs of two donated bodies from the Faculty of Medical and Health Sciences of the University of Auckland under the Human Tissue Act 2008. Donors had no recorded history of knee pain or surgery. Specifications of donated bodies are listed in Table 1. Gender is a crucial factor for in vivo studies of knee implants since, for example, OA is more prevalent in females. For this study and its outcomes, however, donor gender was not significantly important, so the best available specimens were selected. As this is a proof of concept study, two specimens only were used.

**Table 1:** Specifications of donated bodies

Specimen No.	1	2
Gender	Male	Male
Age	64	92
Height (cm)	155	183
Weight (kg)	88	80

Before making any incisions and whilst the knee specimen was intact, MR images were acquired with a Siemens® 3 T MAGNETOM® Skyra (Siemens Medical Systems, Erlangen, Germany) scanner located in the Centre for Advanced Magnetic Resonance Imaging at the University of Auckland. The MRI was acquired to develop a 3D model of the knee joint and design the implant using that model. The knee specimen was imaged using a 15 channel transmit/receive knee coil. Data acquisitions included at least 10 cm above and below the tibial plateau. Different sequences of imaging were acquired in order to facilitate accurate segmentation of tissue. Imaging of each sequence took approximately 5 min and imaging parameters of the sequences are as follows:

- T1 3D vibe Gradient echo sequence: Matrix 320 × 320, Field of view 200 × 200 mm, Slice thickness 0.6 mm, TE 3.79, TR 10.8, Flip angle 10, 1 average.
- T1 3D vibe Gradient echo sequence with fat sat: Matrix 320 × 320, Field of view 200 × 200 mm, Slice thickness 0.6 mm, TE 5.39, TR 10.8, Flip angle 10, 1 average.
- T2 3D Gradient echo sequence: Matrix 320 × 320, Field of view 200 × 200, Slice thickness 0.6 mm, TE 5, TR 14.1, Flip angle 25, 1 average.

**Fig. 1:**
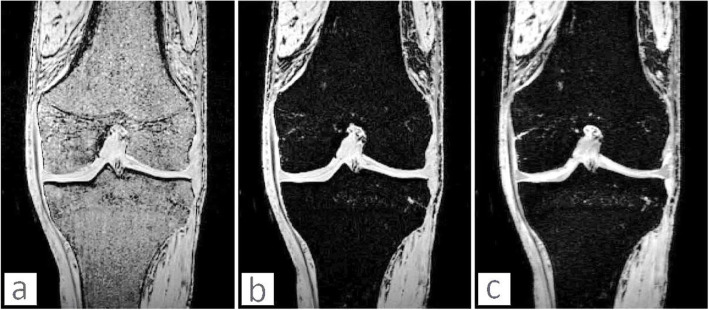
Acquired MRI sequences: **a** T1 3D, **b** T1 3D with fat saturation, **c** T2 3D

The 3D T1, T1 with fat saturation, and T2- weighted images sequences were acquired using identical matrix and resolution as this facilitated segmentation by allowing superimposition of the datasets. The 0.6 mm almost-isotropic resolution facilitated accurate segmentation of the complex structures, essential for the 3D modelling. The anatomy was manually segmented using Amira® software (FEI Visualization Sciences Group, Burlington, MA, USA). Segmentation was based on the image grayscale intensity of the three imaging datasets (Fig. 1), and a priori knowledge of knee structure.

**Fig. 2:**
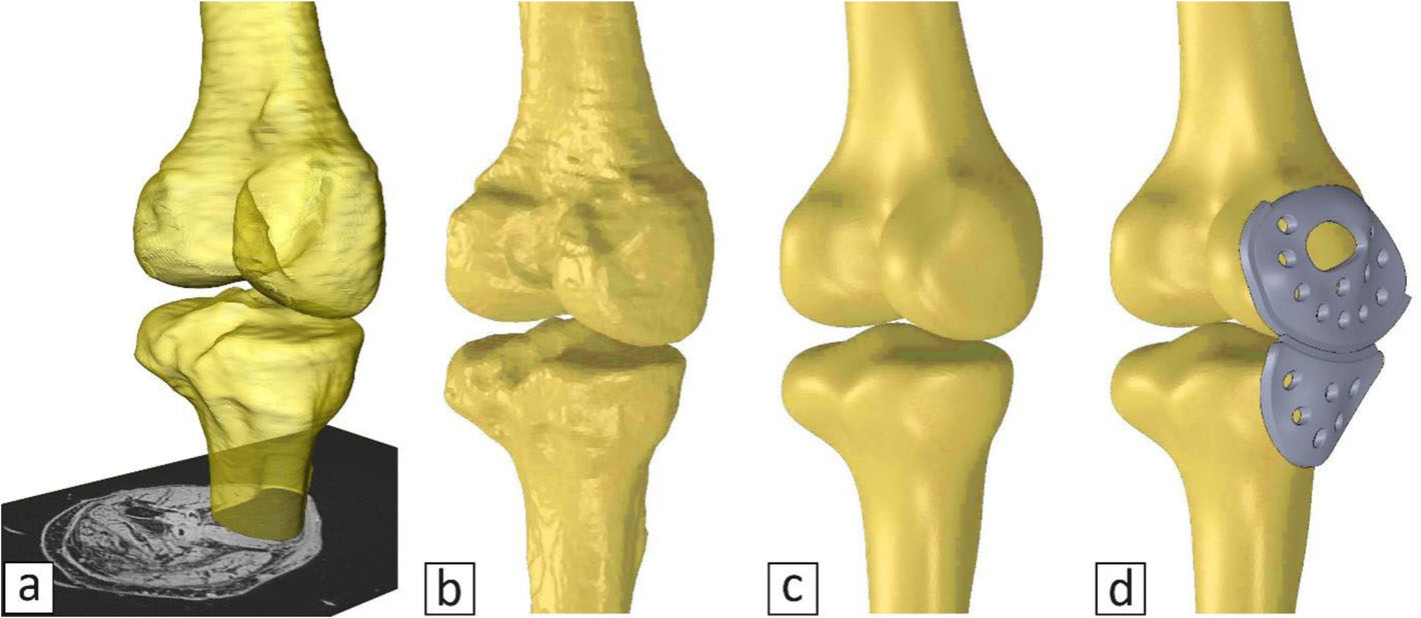
**a** 3D surface rendered model (Amira software), **b** bones before smoothing, **c** smoothed bones using Meshmixer®, **d** knee bones with the implant

Taking advantage of the aforementioned MRI sequences, and switching between them during the segmentation process, the bones were segmented precisely. A semi-transparent surface rendering of the femur and tibia using Amira is shown in Fig. 2a.

After segmentation, STL files of the model were exported (Fig. 2b) and smoothed using Meshmixer software (Fig. 2c). The smoothed model was then imported to SolidWorks® (Dassault Systemes) in order to design the implant to precisely fit the medial side of the model (Fig. 2d). This allows for compatibility between the implant and joint in terms of conformity and curvature of the femoral and tibial heads on the medial side. This is particularly helpful for accurate placement and fit of the implant on the patient’s femur and tibia.

**Fig. 3:**
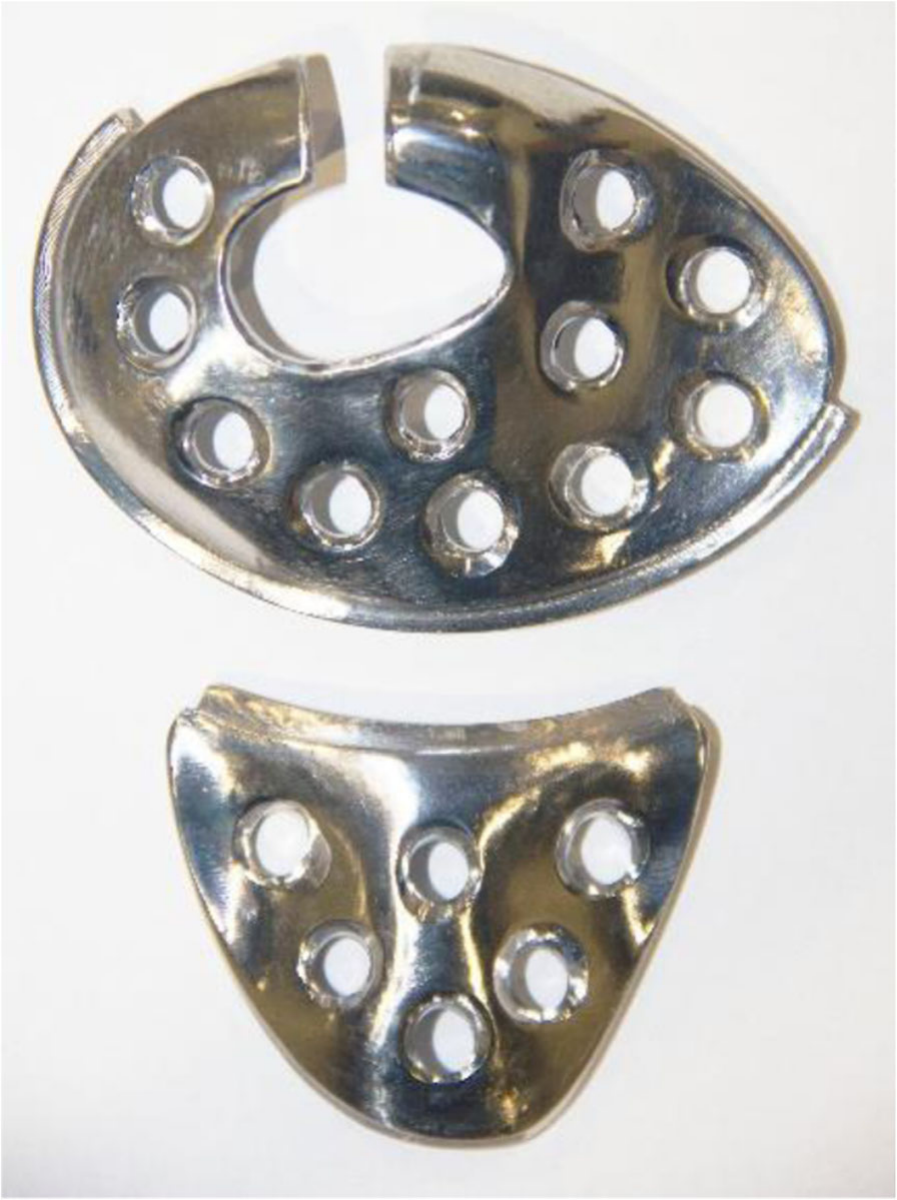
A prototype of the implant manufactured by CNC machining. (Upper part is the femoral component of the implant, and lower one is the tibial component)

This implant is a patient-specific implant, so the best available manufacturing technique for this purpose would be additive manufacturing, in particular Electron Beam Melting (EBM) [13]. However, for this study CNC machining was used, due to the lack of access to an EBM machine. Nowadays, both of these methods are used to produce knee implants. A machined prototype of the implant is shown in Fig. 3.

### Cadaver experiments

**Fig. 4:**
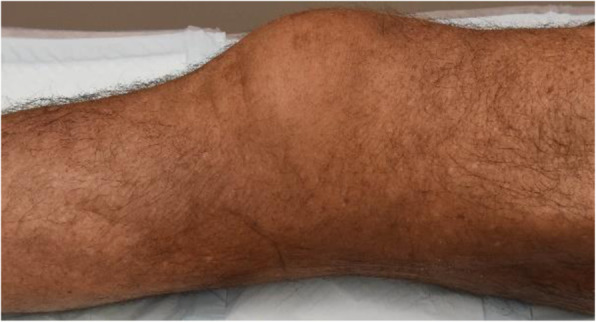
Embalmed specimen No. 1

The complete leg was embalmed before specimen preparation. Topp et al. [24] reported that embalmed and fresh frozen bones showed similar characteristics during mechanical testing and suggested the use of embalmed cadaver specimens as a safe option for testing of orthopaedic devices. For the embalming process, 7 L Dodge® (Dodge Co., Cambridge, MA) anatomical arterial fluid along with 1 L Plasdopake, a tracer dye, which allows embalmer to see evidence of distribution, were used. Hot water (3 L) was added to drive the embalming fluid to the tissue [20, 22]. The knee joint was then isolated from near the middle of the femur to the middle of the tibia as shown in Fig. 4.

Cadaver experiments in this study were divided into two parts: (i) investigating the implant attachment procedure from the surgical point of view (without using any special tool), contact of the femoral and tibial parts at different flexion angles, and the effect of the implant on the joint space in the medial side using X-ray imaging. (ii) comparing the contact pressure in the tibiofemoral joint before and after attaching the implant in the fully extended knee position experimentally.

#### Surgical procedure

Specimen No. 1 had small joint osteophytes which were irrelevant for this part of the study. The device is for early-onset knee OA so specimens with established OA were not tested. Surgery was performed by a knee surgeon in order to determine the limitations, requirements and any specific surgical considerations for the future development of the implant or related tools. Initially, AP and lateral X-rays of the specimen were taken, and an incision was made on the medial side of the knee joint to expose the Medial Collateral Ligament (MCL). After exposure of the ligament, the implant was attached to the femur and tibia using four screws for the femoral plate and three screws for the tibial plate, without damaging the MCL. After attaching the implant, X-rays were taken again in order to determine the difference in the joint space induced by the implant. A surgeon then examined the contact of the femoral and tibial components at different flexion angles. Detailed information regarding the load sharing mechanism in this implant is discussed in a study by Saeidi et al. [16].

#### Contact pressure measurement

For the contact pressure measurements, specimen No. 2 was used. Before conducting any experiments, all of the soft tissue except for cruciate and collateral ligaments were removed because there is no muscle force in a cadaver specimen. It should be noted that the implant design allows to protect the capsular ligament and there is no need to remove any lateral structure. Loading scenarios were applied using an Instron machine with a 10 kN load cell in the fully extended knee position. Assuming 80 kg as average body weight, three loading forces from one to two times body weight were used for the experiments. Depending on the activity and joint angle, the knee joint can undergo much higher pressure but, for this preliminary study, only the mentioned loads were applied to the joint; experiments were conducted using this specific setup to compare the contact pressure in the knee joint before and after attaching the implant and not necessarily to exactly mimic the range of real-life loading conditions, as this needs a more advanced test rig which can be done in the later stages of implant development.

**Fig. 5:**
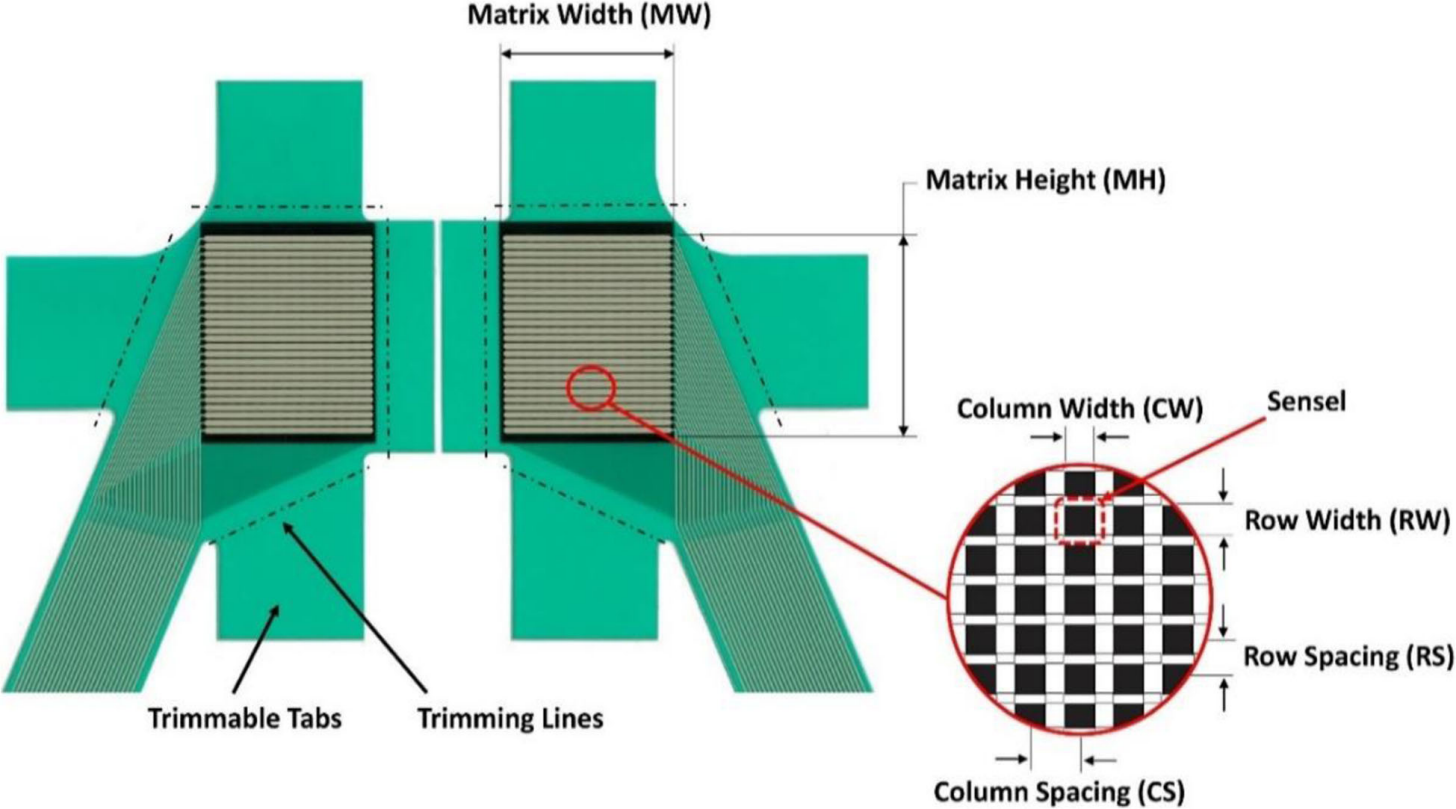
Tekscan pressure mapping sensor 4000 and a magnified sensing matrix, used for the experiments

Each load was applied three times and the average was reported for each test condition. The knee joint was mounted on the machine using fixtures and the contact pressure in the tibiofemoral joint was measured before and after attaching the implant using a Tekscan® pressure mapping sensor model 4000–1500 (Tekscan, Inc., Boston, MA), which can measure up to 10 MPa pressure. As shown in Fig. 5, intersections of conductive paths, which are covered by a pressure-sensitive ink, form a sensing matrix. This sensor is very thin (0.1 mm) and flexible, and so can be used in the narrow joint space, in which the bone surface is not flat. Specifications of the sensor are listed in Table 2.

**Table 2:** Tekscan pressure mapping sensor 4000 specifications [23]

MW (mm)	MH (mm)	Columns			Rows			Total No. of Sensels	Sensel Spatial Resolution
		CW (mm)	CS (mm)	Qty.	RW (mm)	RS (mm)	Qty.		
27.9	33.0	0.8	1.3	22	1.0	1.3	26	572	[sensel per sq-cm] 62.0

**Fig. 6:**
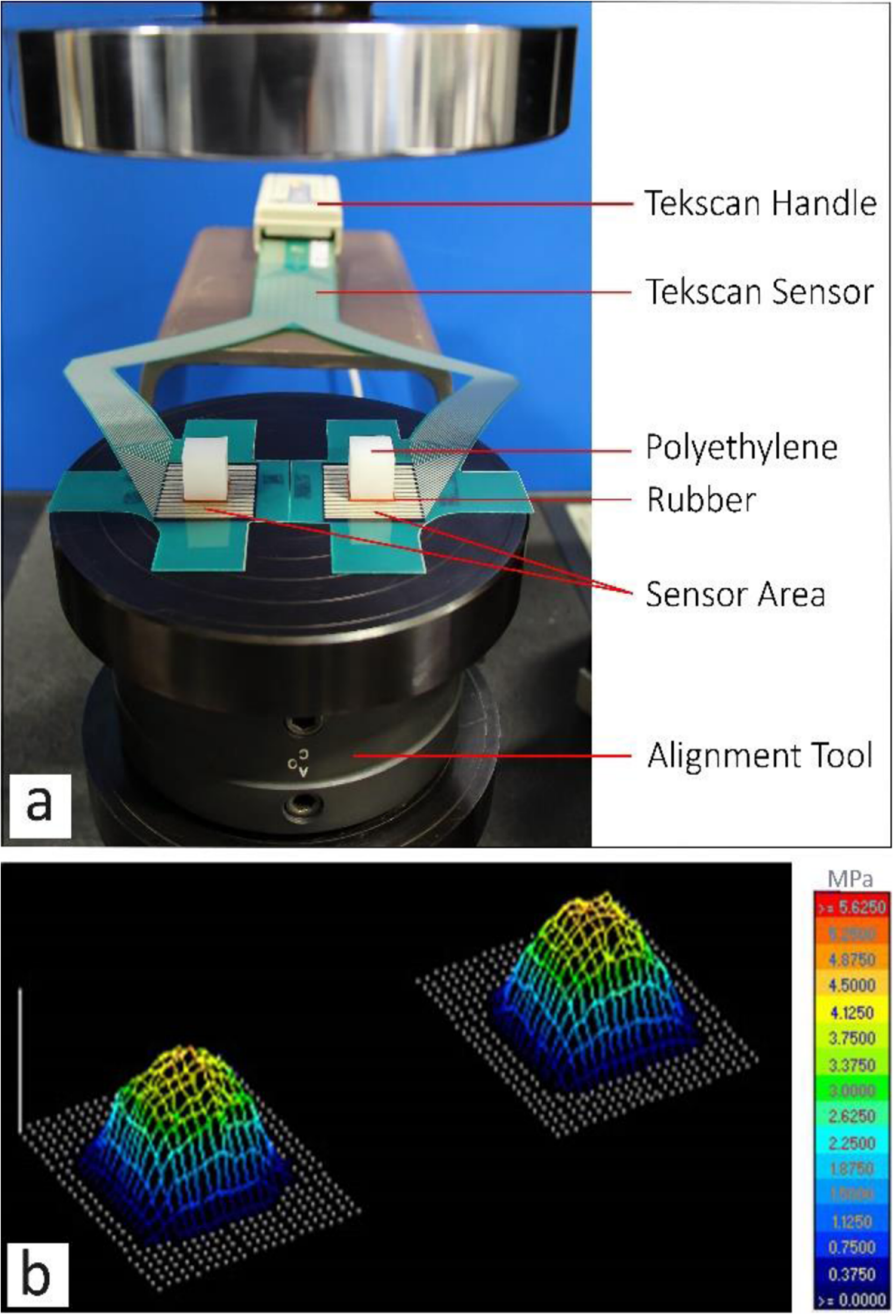
**a** sensor calibration setup, **b** graphic representation of pressure distributions during the calibration process

Positioning and fixing the sensor in the joint is crucial to accurately record and compare the pressures obtained from experiments without and with the implant. Care was taken during placement of the sensor to avoid damaging or wrinkling the sensor, and to ensure a correct fitting of the sensor on the soft tissue. In order to fit the sensor properly, most of the tab around the sensing area needed to be trimmed before insertion. In order to have an accurate understanding of how the implant influences the contact pressure at both compartments, the applied load should be fully transmitted through the sensor. Therefore, the menisci were removed in order that the sensor covered the whole contact area. The sensor was connected to the handle (as shown in Fig. 6a) to begin measurements once all the preparations were completed. Experiments were performed with two different set-ups: without and with the implant. Prior to the experiments, the sensor was preconditioned in order to improve repeatability, by loading and unloading five cycles up to 110% of the maximum load.

**Fig. 7:**
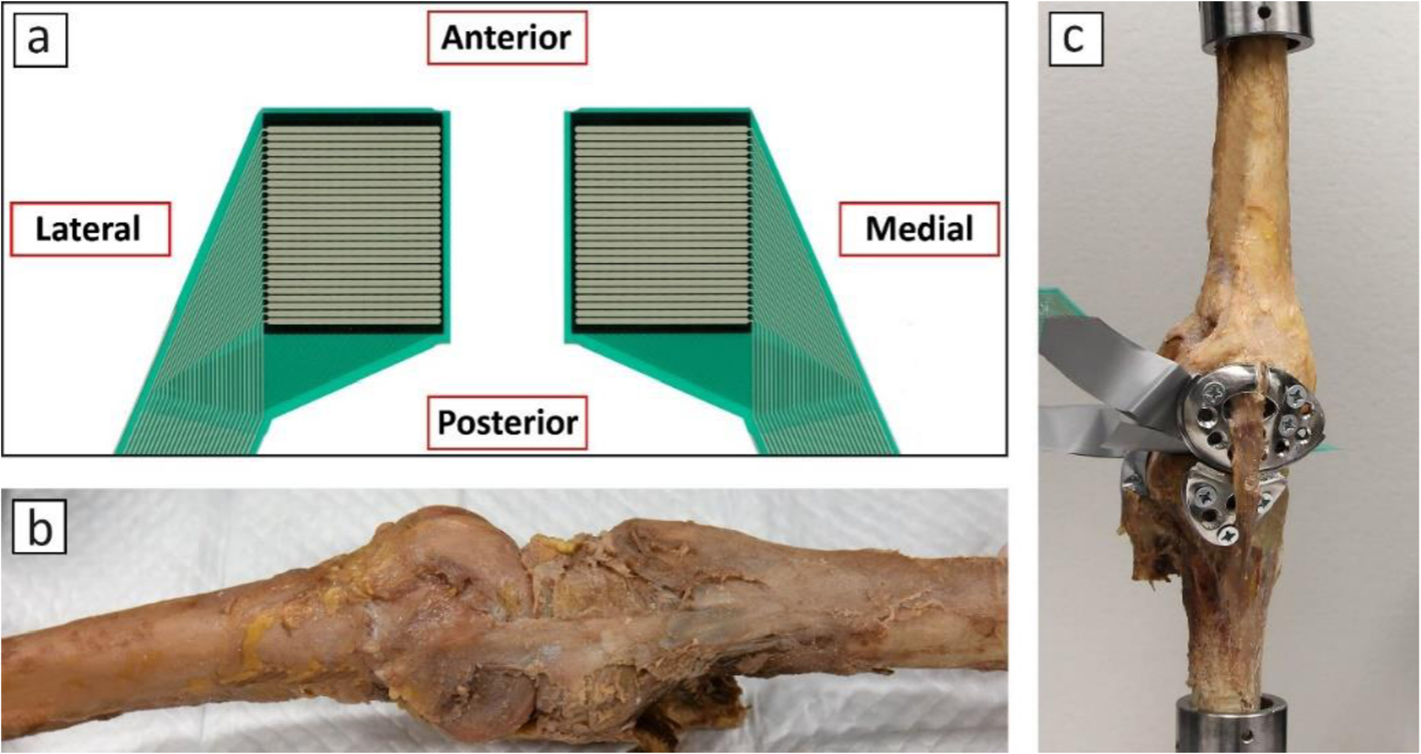
**a** direction of the sensor in the joint during experiments, **b** medial view of the specimen without implant, **c** experimental setup with implant

Because the knee joint was a cadaver specimen, non-surgical grade screws were used to attach the implant to bones. As mentioned, no ligament is sacrificed in this method and the attachment point of the MCL to the femur was considered during the design procedure of the implant. The medial side of the specimen is shown in Fig. 7b.

**Fig. 8:**
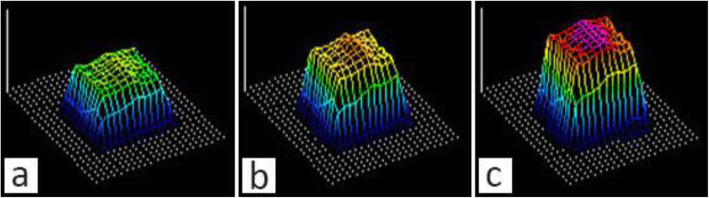
Example of the pressure contour at different sensitivity levels: **a** low sensitivity (S17), **b** optimal sensitivity (S19), **c** oversaturation (S21)

During the experiments, raw data was collected and the calibration process was conducted based on the collected data after the experiments. The sensitivity level should be the same for the calibration process and the experiments. To adjust the sensitivity, the sensor was placed in the experimental setup and loaded up to 90% of the maximal intended experimental load. The saturation level was then adjusted in such a way so as not to observe oversaturation (purple colour). Three different sensitivity levels are demonstrated in Fig. 8 with S19 (sensitivity level adjusted in the I-Scan™ software) being an optimum sensitivity based on the maximum load.

**Fig. 9:**
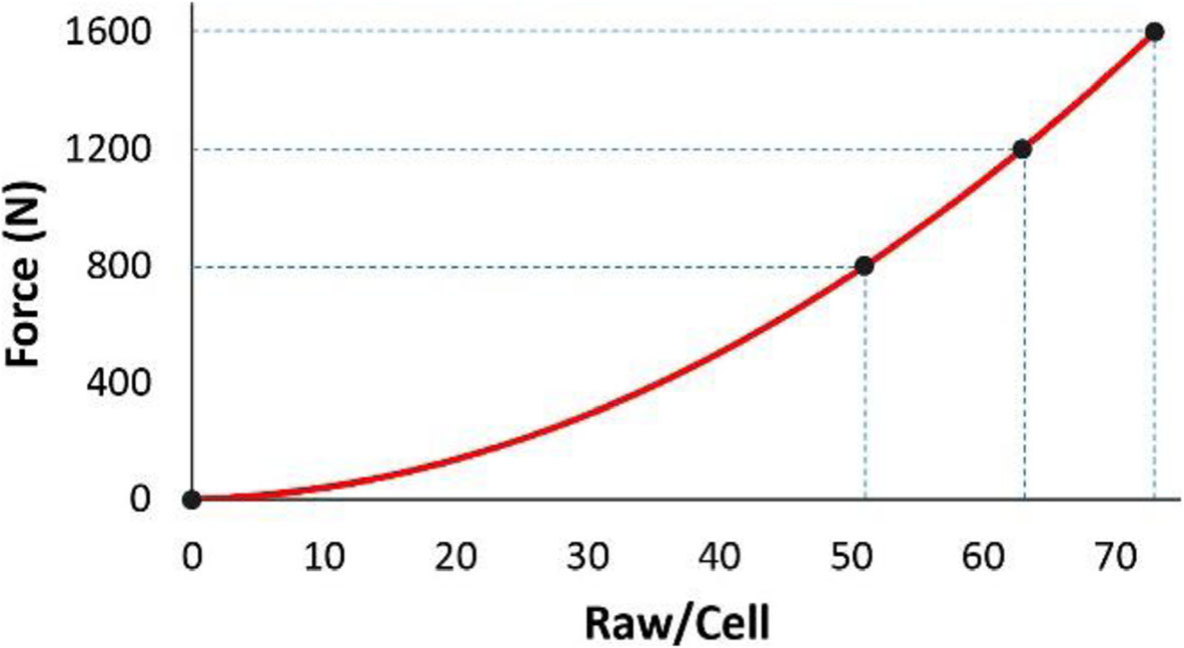
Calibration curve generated by the I-Scan software

To calibrate the sensor, initially the average raw pressure of the collected data during experiments was measured for all loading conditions. To ensure an accurate calibration over the whole loading range, a multipoint calibration was performed due to the non-linear correlation between the applied load and the measured raw data from the sensor. Therefore, a 3-point calibration was conducted using three average raw values previously collected during the experiments [26]. I-Scan™ software was then used to create a calibration curve as shown in Fig. 9.

The calibration was performed using the setup shown in Fig. 6a, in which polyethylene simulates the cartilage surface and a rubber layer ensures uniform pressure distributions on the sensor [26]. An alignment tool at the bottom of the experimental setup was introduced in order to balance the axial load at both sides of the sensor. Pressure distributions under a 1600 N load during the calibration process are shown in Fig. 6b.

## Results

### Surgical procedure

**Fig. 10:**
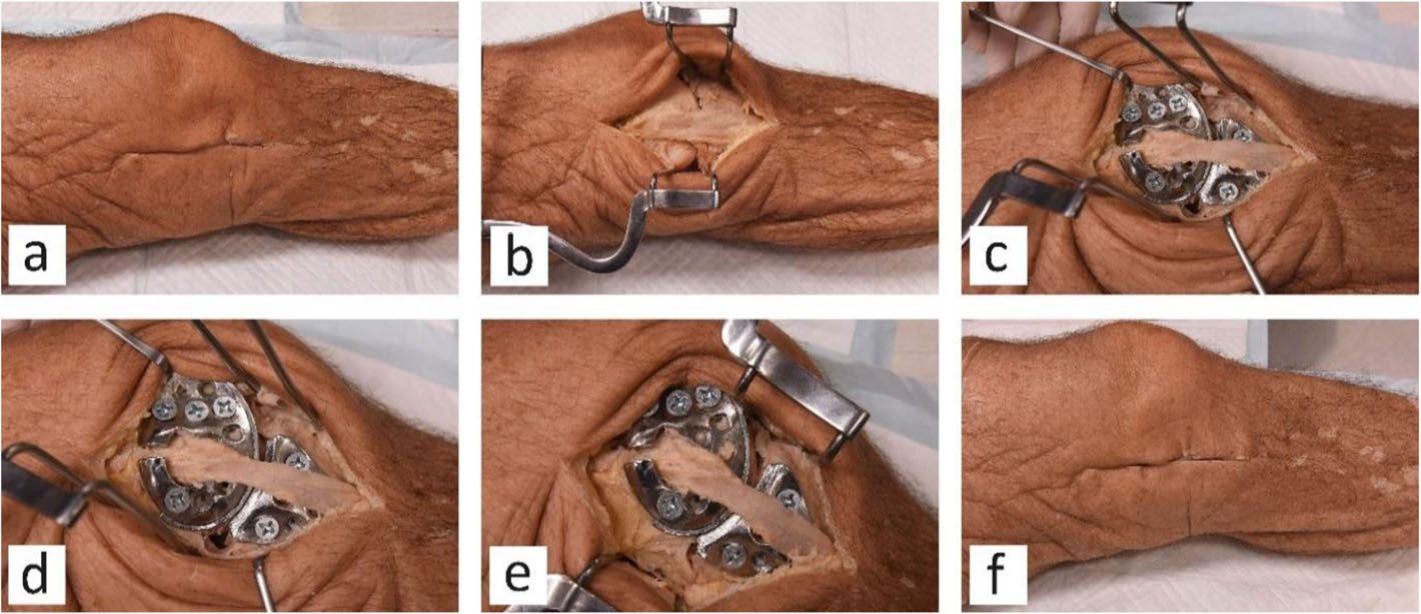
**a** incision made in the medial side of the specimen, **b** exposed MCL, **c** joint at 0°, **d** joint at ~ 30°, **e** joint at ~ 60°, **f** closed incision after attachment of the implant

After making an incision (Fig. 10a), exposing the MCL (Fig. 10b) and attachment of the implant, the knee joint was examined at different flexion angles as shown in Fig. 10c – e. Finally, the incision was closed as shown in Fig. 10f.

**Fig. 11:**
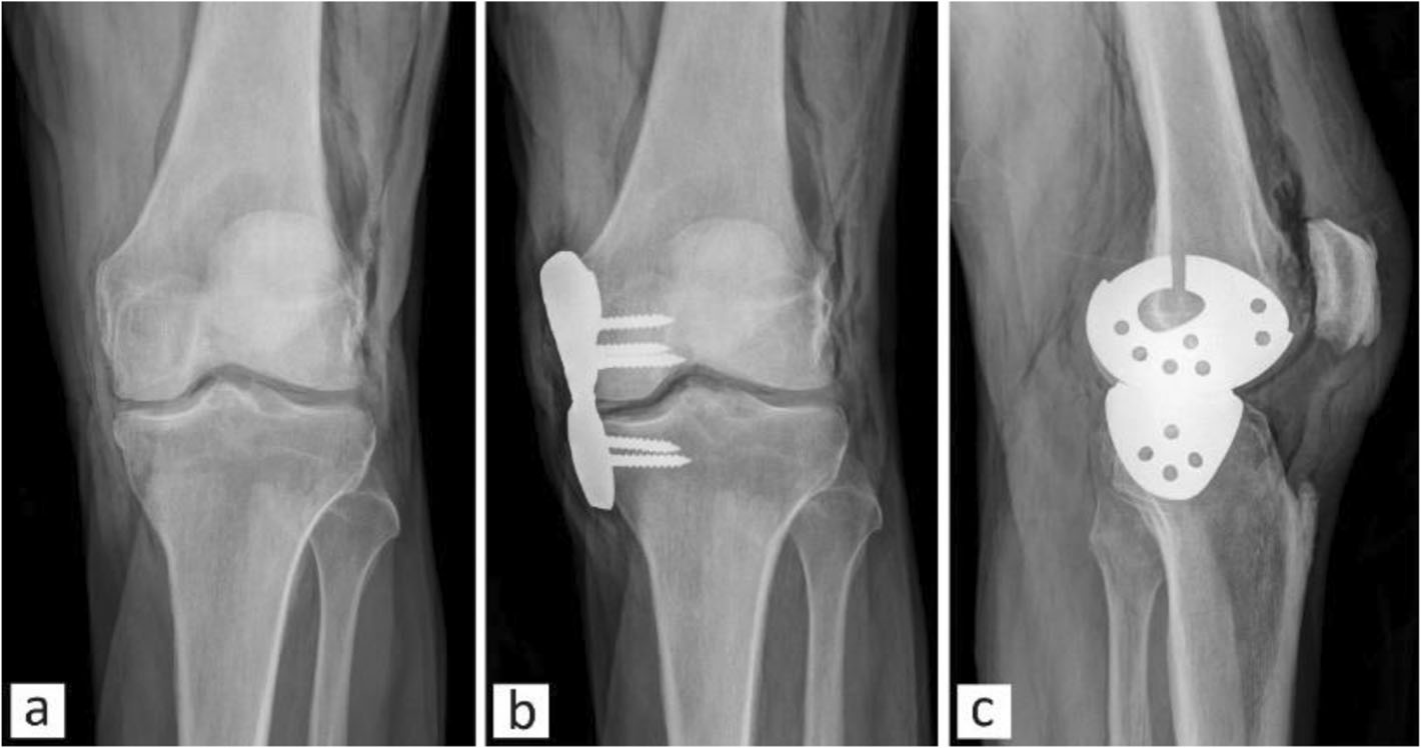
X-ray of the knee joint: **a** AP without implant, **b** AP with implant, **c** lateral with implant

An AP X-ray of the knee joint conducted when the specimen was intact is shown in Fig. 11a. AP and lateral X-rays of the joint after introducing the implant are shown in Fig. 11b and c, respectively.

### Contact pressure

As previously described, cadaver experiments were performed under different loading scenarios and results were recorded using a pressure mapping sensor. As shown in Fig. 7a, the top and bottom of the pressure map show the anterior and posterior sides of the joint. After conducting the initial experiments, the implant was introduced to the joint. Ideally, joint space in the medial side should be slightly increased before attachment of the implant in order to reduce the stress in the damaged cartilage. To do so would subject the knee joint to valgus strain during a real surgery; however, doing so during the experiment could lead to a different experimental setup and consequent error. Therefore, the implant was attached to the specimen while the joint was still mounted on the Instron machine.

**Fig. 12:**
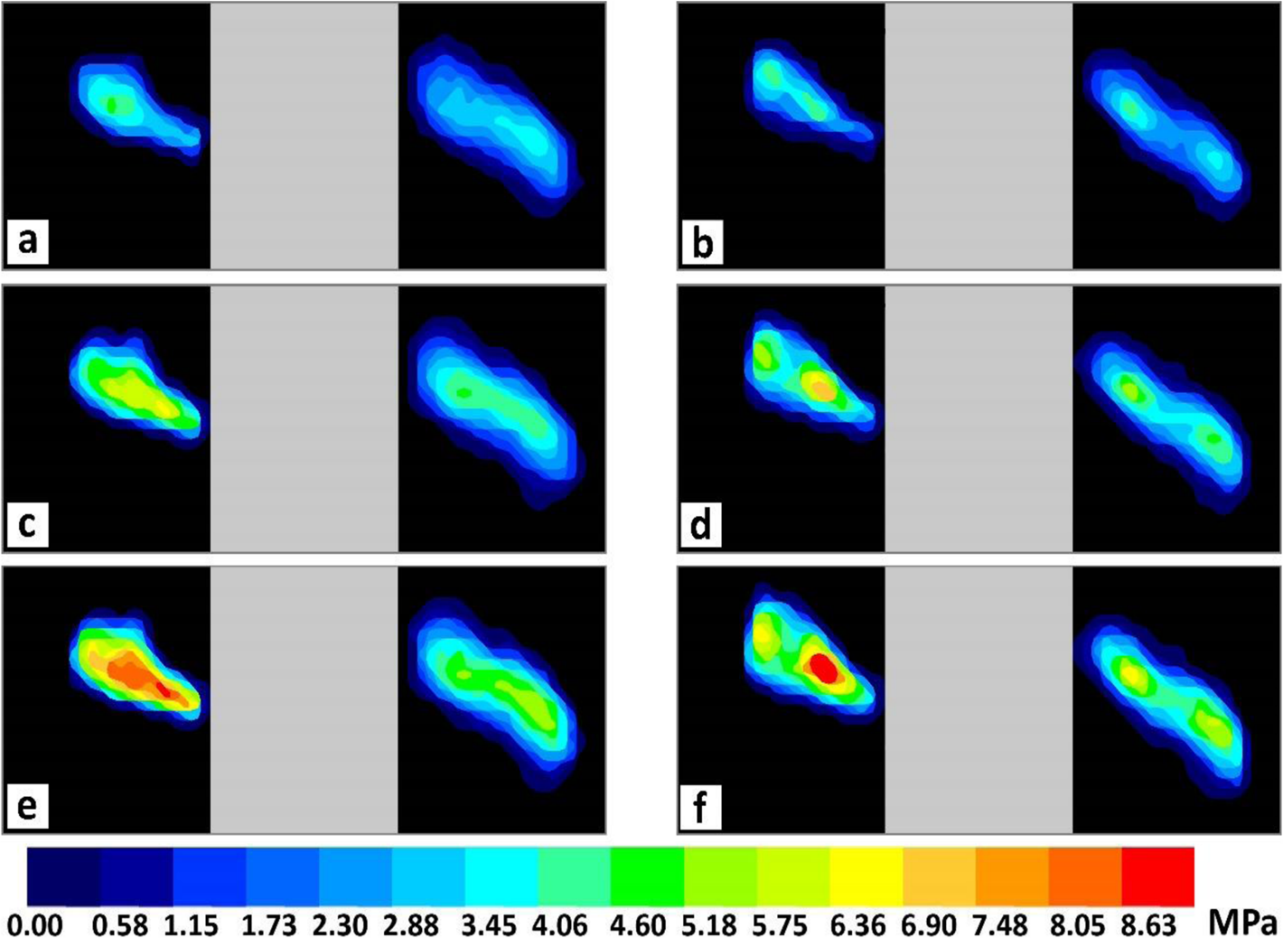
Contact pressure distributions in the knee joint under different loads, **a** 800 N without implant, **b** 800 N with implant, **c** 1200 N without implant, **d** 1200 N with implant, **e** 1600 N without implant, **f** 1600 N with implant

Pressure distributions under each loading condition for the experiments without and with the implant are shown in Fig. 12. The orientation of the sensor in the joint is shown in Fig. 7a, i.e. the right and left sides of each image in Fig. 12 show the pressure distribution in the medial and lateral compartment, respectively.

## Discussion

### Surgical procedure

As shown in Fig. 10a, the incision made to attach the implant is smaller than the typical incision required for a standard joint replacement. It should be taken into account that the actual incision during surgery could well be smaller than what was made during the experiments, as a good exposure of the implant was required in this study in order to take photos and measurements. One of the advantages of this implant is that the anatomy of the joint is not disrupted during the procedure. Indeed, as shown in Fig. 10, the MCL was not sacrificed or damaged, nor were the bones modified, thus the implant can be easily removed if required.

The joint moved smoothly during flexion and extension without any resistance due to the implant. This was a visual evaluation; further research will need to be conducted in the future, such as measuring kinematics before and after attachment of the implant to make sure they are unaltered, and measuring pressure in order to show whether any changes have occurred throughout the flexion arc after implantation. In the fully extended position of the knee joint, a distributed load was observed between the articulating surface of the femoral and tibial components (Fig. 10c), while at other angles a more concentrated load was observed (Figs. 9e and 10d). Given the fact that point loads at metal-on-metal contacts could lead to wear, and metal debris and complications such as metallosis, it should be emphasised here that this implant is a prototype made of stainless steel. Material selection and long-term survival rates of the implant bearing surfaces was not the focus of this study and is being investigated in a separate project. The contact points between components for the final design is likely to be a metal (Ti-6Al-4 V or CoCr) on polymer (Ultra High Molecular Weight Polyethylene), similar to that in common TKA implants. The above risk factor as well as some other issues, for example tissue irritation around the implant, will be investigated in a further in vivo study using the final version of the implant.

X-ray of the knee joint was conducted to determine the impact of the implant on the joint space. As shown in Fig. 11, the gap between femur and tibia increased after introducing the implant. For this experiment, a slight valgus deflection (~ 2 mm at the interface between the implant parts before fixation) was manually created and the implant was then fixed to the joint; a tool or specialised equipment will be developed for actual surgery, to create a measurable deflection based on preoperative radiographs. The X-rays also clearly showed the position of the femoral and tibial implant components on the bone and on each other (Fig. 11b and c). Both parts of the implant were positioned on each other as expected and no gap was observed between them in either the AP view or lateral view.

### Contact pressure

**Fig. 13:**
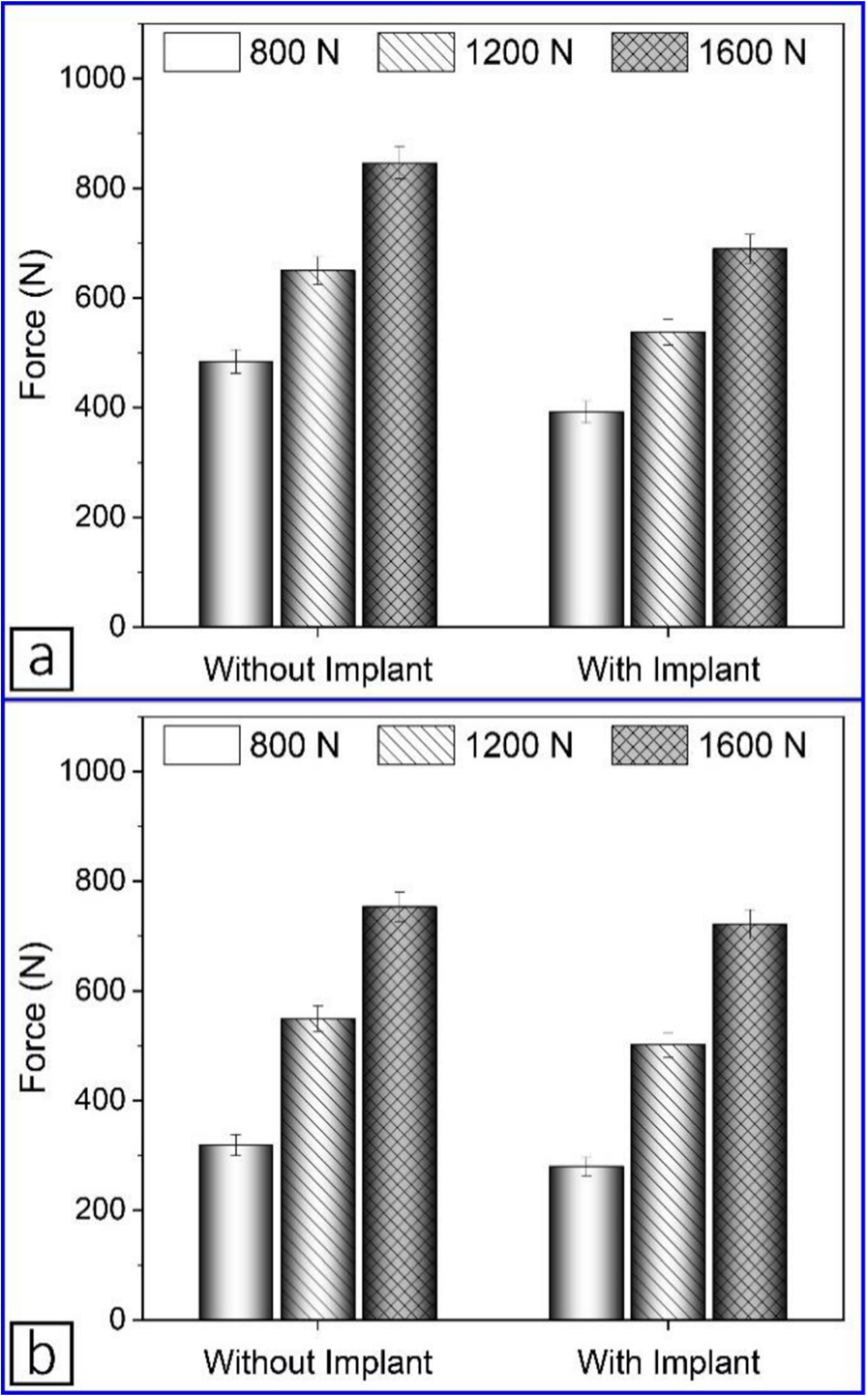
Force in the knee compartment without and with implant: **a** medial, **b** lateral

Measured forces in the medial and lateral compartments of the knee joint are shown in Fig. 13a and b, respectively. The implant was attached to the medial side so it had a greater effect on this side, in terms of reducing the force. As mentioned, the implant was attached to the specimen while it was mounted on the machine without creating a valgus deflection; otherwise more load reduction would have been observed.

**Fig. 14:**
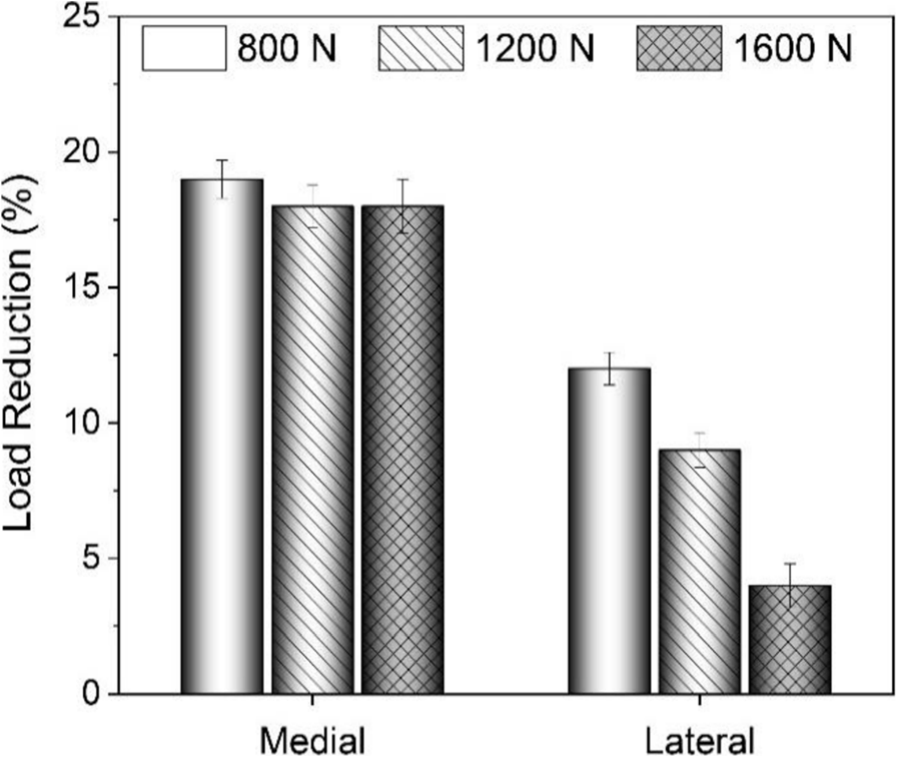
Load reductions in both compartments after attachment of the implant

The percentage load reductions in the medial and lateral compartments after attachment of the implant are shown in Fig. 14. It was observed that under different loading conditions, the implant reduced the load going through the medial side by approximately 18%. Increasing the applied load on the specimen from 800 N to 1600 N caused the percentage of load reduction in the lateral side to decrease approximately from 12% to 4%. This implies that increasing the load led to abduction of the joint and the transmission of a higher percentage of the applied load through the lateral side. According to the mentioned results, the implant was effectively reducing the load going through the medial side.

**Fig. 15:**
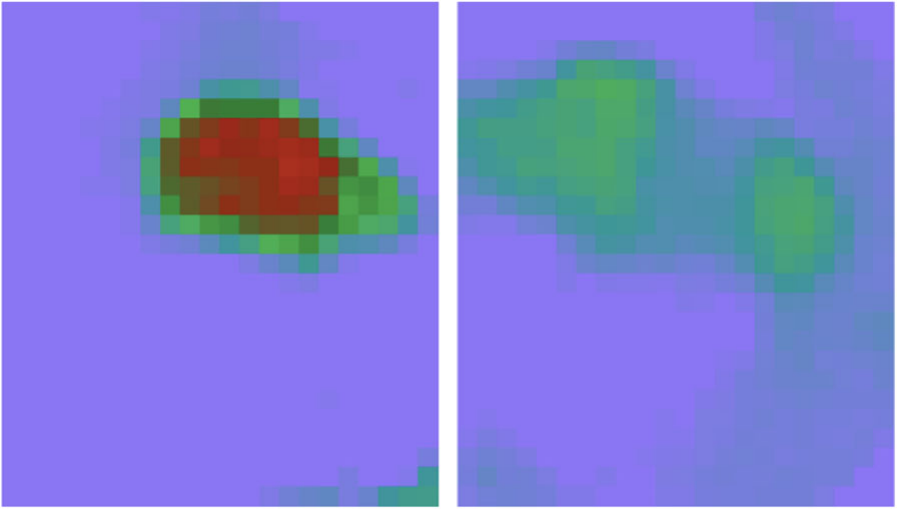
Measured contact pressure in the knee joint under 1000 N by Tekscan sensor [4]

The experimental results were compared with similar studies: Pressure distributions were compared with cadaver research conducted by Fojtik [4] on the influence of Lateral Meniscus Posterior Root Avulsions (LMPRA) and Meniscofemoral Ligament (MFL) deficiency on contact mechanics of the tibiofemoral joint. She compared pressure contours of an intact knee joint, a joint with LMPRA, and a knee joint with LMPRA and deficient MFL. Pressure contour patterns were similar for the two aforementioned conditions; pressure distributions for the latter condition are shown in Fig. 15, and show the distinctive pressure field on the tibial plateau caused by the femoral cartilage. Pressure contour patterns in experiments performed by Fojtik (Fig. 15) are similar to what was observed in the present study (Fig. 12). Similarly, higher peak contact pressure in the lateral compartment was observed in both studies, due to a smaller contact area on this side compared to the medial side.

**Fig. 16:**
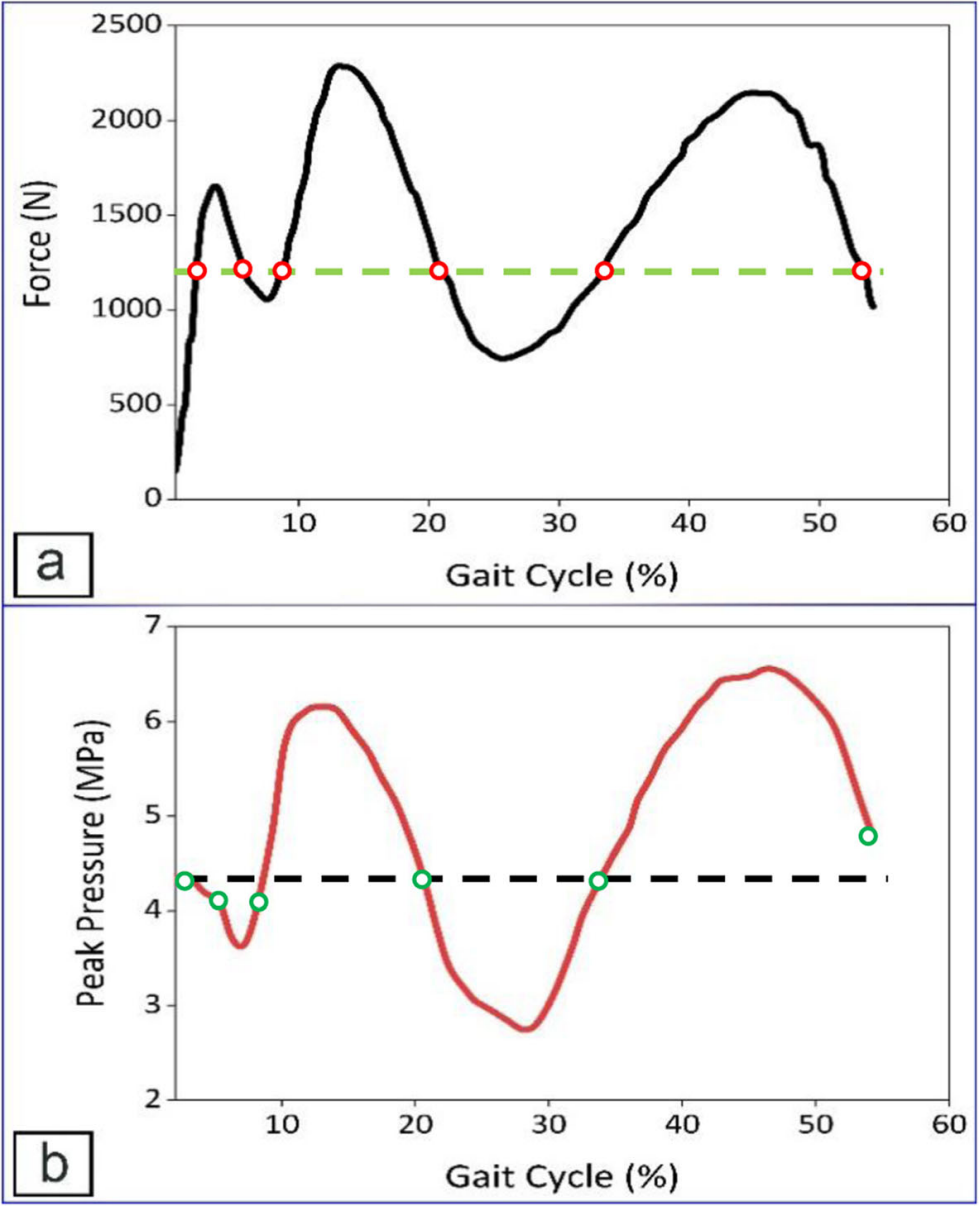
**a** input of the knee simulator [25], **b** measured peak contact pressure in the medial side by Tekscan sensor [25]

Peak contact pressure in the medial side was also compared with what was reported in a cadaver study conducted by Wang et al. [25]. They used a dynamic knee simulator in order to simulate the gait cycle and measure the contact pressure in the knee joint for different conditions, i.e., intact knee joint and meniscectomy. Due to the menisci removal in the present study, the meniscectomy condition was selected for comparing results. The role of the meniscus is mainly to distribute pressure over the tibial plateau; because the experiment aimed to measure the force going through each compartment, removing the menisci did not considerably affect the outcome of the current study. The input of the dynamic knee simulator and peak contact pressure reported by Wang et al. [25] are shown in Fig. 16a and b, respectively.

Applied loads for the present study were 800, 1200, and 1600 N, and they were applied at zero degree flexion. In the research conducted by Wang et al. [25], these loads were applied on the joint at different angles during the gait cycle. For instance, 1200 N (green dash-line in Fig. 16a) was applied on the knee joint six times during one gait cycle (red points). The percentage of the gait cycle was read from the input graph for all six times and the equivalent peak pressure for each percentage was read from Fig. 16b (green points). The mean value and standard deviation for the six peak pressure values were around 4.3 (black dash-line in Fig. 16b) and 0.25 MPa, respectively. Given that the peak pressure does not significantly differ for any given load at various percentage of the gait cycle, the X-axis of the peak pressure graph does not start from zero (Fig. 16b), and also the least flexion angle occurs at around 40% of the gait cycle [25], the 800, 1200, and 1600 N were applied at 24%, 34%, and 38% of the gait cycle, respectively, and the equivalent pressures were 3.2, 4.3, and 5.4 MPa, respectively. For the same loads in the present study, measured peak contact pressures were 3.7, 4.6, and 5.5 MPa, respectively, relatively close to what was reported by Wang et al. [25].

### Limitations

In this proof of concept study, only two specimens were used. A thorough cadaver study with many more specimens and appropriate statistical analyses should be conducted in the future. Other devices, such as the Atlas® system, should also be tested and results compared with those of the studied implant.

## Conclusions

The concept of an extra-articular load-sharing implant for osteoarthritic knees was tested in this preliminary study. The implant was initially attached to the medial side of a cadaver knee joint and examined at different flexion angles without modifying the bones or sacrificing and/or damaging the MCL. It was observed that after attachment of the implant, the joint moves smoothly without any resistance caused by the implant. According to AP and lateral X-rays before and after introducing the implant, joint space in the medial compartment increased and both parts of the implant were positioned properly on each other.

Contact pressure in the tibiofemoral joint was also studied under different loading conditions. It was observed that applied forces on both compartments decreased after attaching the implant; however, the force going through the medial side was decreased more than that of the lateral side, because the implant was attached to the medial side. Peak contact pressures were higher in the lateral side, because of the smaller contact area in the lateral compartment as compared to that in the medial side. It was also observed that, after attachment of the implant and under different loading conditions, the load was reduced by approximately 18% in the medial side. On the other hand, by increasing the applied load on the joint from 800 N to 1600 N, the percentage load reduction decreased from by 12% to by 4% in the lateral side, due to abduction of the knee joint.

The results of the current study show that this implant can effectively increase the joint space on the medial side and reduce the load going through that side without sacrificing or damaging any of the hard or soft tissues, indicating some promise for this type of solution in the treatment of knee osteoarthritis, and points to the merit of a more comprehensive program of testing followed by in vivo studies.

## Data Availability

Not applicable.
